# Metal removal and associated binding fraction transformation in contaminated river sediment washed by different types of agents

**DOI:** 10.1371/journal.pone.0174571

**Published:** 2017-03-28

**Authors:** Hong Wang, Tongzhou Liu, Shuai Feng, Weihua Zhang

**Affiliations:** 1 School of Environmental Science and Engineering, South University of Science and Technology of China, Shenzhen, P. R. China; 2 Engineering Innovation Center (Beijing), South University of Science and Technology of China, Beijing, P. R. China; 3 Harbin Institute of Technology Shenzhen Graduate School, Shenzhen Key Laboratory of Water Resource Utilization and Environmental Pollution Control, Shenzhen, P. R. China; 4 School of Environmental Science and Engineering, Sun Yat-sen University, Guangzhou, P.R.China; 5 Shenzhen Research Institute of Sun Yat-sen University, Shenzhen, P. R. China; University of Vigo, SPAIN

## Abstract

In ex-situ washing, HCl, EDTA and H_2_O_2_ solutions can effectively extract heavy metals in river sediment. Nevertheless they often target different sediment components, possibly transforming metal species into more bioavailable and hence toxic ones. This study, in batch settings, investigated the influences of different types of washing agents (i.e. HCl, EDTA and H_2_O_2_) on metal (i.e. Cu and Zn) removal from contaminated river sediment, destroy or dissolution of sediment components, and transformation of metal fractions during chemical washing treatment. Additionally, bioavailability of these metals left in the washed sediment was assessed. Results showed that HCl obtained the highest Cu and Zn removal through destroying the reducible, oxidizable and residual sediment components. Meanwhile, it transformed metal fractions to acid extractable one, resulting in an increase in metal bioavailability. Thus, the feasibility of washing with HCl for sediment remediation shall be reconsidered due to the caused high metal bioavailability. EDTA was capable of removing metals via direct complexation of labile metal species and indirect dissolution of reducible and oxidizable sediment components, where the transformation of corresponding metal binding fraction may occur. H_2_O_2_ obtained the lowest total Cu and Zn removal, but it preferentially removed the oxidizable metal species by oxidizing sulfides in the sediment. The bioavailable levels of Cu and Zn in the sediment washed by EDTA or H_2_O_2_ seemed not increase. To maintain a good balance between labile metal species removal and avoiding increase of metal bioavailability, EDTA and H_2_O_2_ are promising additives for metal removal by sediment washing.

## Introduction

Heavy metal contaminated sediment in rivers those are subject to anthropogenic discharges is a major problem from environmental perspective. Considerable heavy metal pollutants initially generated from manufacturing activities, have been discharged or leaked into the surface waters in the Pearl River Delta of South China. Majority of these heavy metals are first retained onto suspended solids, and then deposited on the river bed, leading to heavy metal enrichment in river sediment. When river environment is disturbed by water currents change (e.g. flooding or tidal intrusion) and anthropogenic disturbance (e.g. river channel maintenance dredging) sediment re-suspension would occur and heavy metals in sediment can release back into the overlying water [1], threatening the aquatic biota. Therefore, it's necessary to develop cost-effective techniques to clean up the heavy metal contaminated sediment.

Similar to soil, there are two remediation strategies for heavy metal contaminated sediment. One aims at immobilizing metals “in situ” by enhancing metal sorption, precipitation and complexation capacity of sediment, or isolating (e.g. active capping) the contaminated sediment to the overlying water, thus reducing the potential mobility or bioavailability of toxic metals. Noteworthy, these techniques are relatively low-cost with simple operations, but the metals in sediment won’t be chemically removed. It’s possible that the caged metals re-enter the water column under certain favorable conditions in long term [2, 3]. The other remediation strategy is to extract or separate metals via washing or flotation from sediment. This technique is usually carried out “ex situ” with a high cost, but it can permanently reduce metal contents and remove most mobile metal species.

Sediment washing is a simple and straightforward ex situ remediation technology, which involves transferring heavy metals from the dredged sediment to the aquatic solution through adding washing agents such as inorganic acid, chelates and oxidants [4, 5, 6, 7]. These additives can facilitate the dissolution, dispersion and mobilization of weakly-bound metals primarily in the forms of exchangeable hydroxides and carbonates [8, 9, 10, 11]. These species usually can coordinate with chelates to form stable and soluble complexes, subsequently detach from sediment. In addition, these additives are also widely reported to be able to destroy or dissolve sediment/soil components, releasing associated non-labile metals [12, 13, 14]. As river sediment typically owns higher contents of humic substrate and clay compared to soil, which often have a strong affinity with heavy metals [10,15, 16, 17], the destroy and dissolution of these sediment components can lead to a substantial metal release. These processes are often divided into two phases: the fast metal destabilization by the attacking of the additives; and the rate-limiting detachment of the associated metals [13, 14, 18]. The latter is a kinetically driven process and usually governs the fate of the destabilized metals. If the detachment is not completed due to the insufficient contact time, it may lead to an increase of metal toxicity and bioavailability due to the formation of weakly-bound metal fractions [19, 20].

The contribution of these two mechanisms depends on heavy metal fraction distribution and the properties of washing additives. It also influences metal removal and associated ecological risk of the washed sediments. However majority of the information addressing heavy metal fraction distribution shift due to chemical washing was primarily obtained from studies on soil remediation. Besides, different metals are often primarily bound to different components of sediment. For example, Cu is widely reported to favorably associate with organic matters [21], while Zn is primarily bound to carbonates and oxides [22]. Consequently, the extent and rate of metal detachment will be controlled by the selective attack of sediment components when the washing agents were added. To provide more information for facilitating better engineering design of sediment washing treatment, the objective of this study is therefore to investigate the influences of different often used types of washing agents (i.e. HCl, EDTA and H_2_O_2_) on the metal removal, destroy or dissolution of sediment components, as well as the transformation of metal fractions in sediment during chemical washing treatment.

## Materials and methods

### The sediment collection and pretreatment

**Fig 1:**
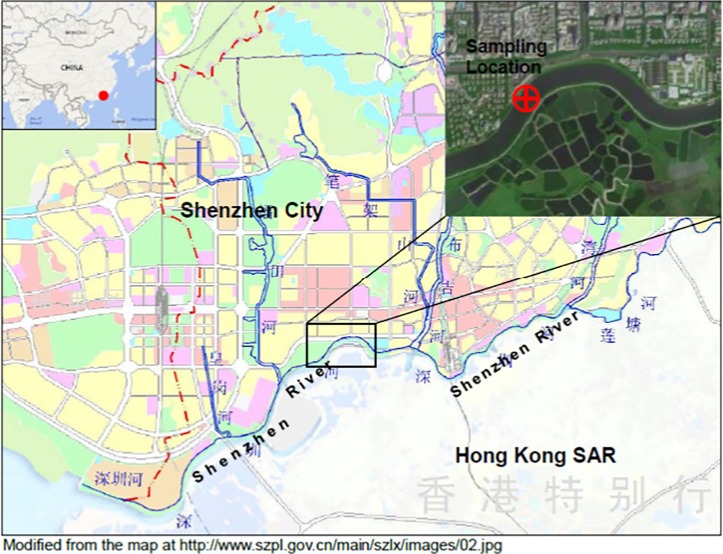
Location map of sediment sampling in Shenzhen River.

The studied sediment was collected from the upper sediment layer 0–0.5 m below the bed of the Shenzhen River (114° 5′ 4″ E, 22° 31′ 55″ N) located in Shenzhen, China, and the sampling location map is illustrated in Fig 1. That sampling site was located in the public land and no specific permission for sediment sampling collection was required. The sampling activity did not involve endangered or protected species, as no such species live there.

The collected sediment was firstly sieved by a 10-mesh laboratory test sieve to remove the coarse stones, twigs, and other debris. Then it was homogenized and air dried in room temperature. The sediment was characterized, and its main properties were presented in our previous study [23]. In that study, it was found that fraction distribution of the metal remained in chemically washed sediment was transformed, possibly leading to enhanced metal leaching extractability. To further investigate the performance of these washing agents (i.e. HCl, EDTA and H_2_O_2_) on different sediment components, with which metal species were associated, the collected sediment was pretreated to remove some metal fractions based on the modified European Community Bureau of Reference (BCR) three step sequential extraction procedure [24]. The sediment treated after step 1, step 2, and step 3 extraction were rinsed with deionized water, air dried, and then named as RF1, RF2 and RF3 samples, respectively. Chemicals used in the extractions and metal fractions left in the sediment are summarized in Table 1. The surface morphology of the original sediment was analyzed using a field emission scanning electron microscope coupled with energy dispersive spectroscopy (SEM-EDS, JEOL JSM-6701F). Its mineral components were analyzed using Empyrean X-ray diffraction (XRD) equipped with the MIMI Jade 6.5.

**Table 1:** Chemicals used in the sequential extractions and metal fractions left in the sediment at different extraction steps.

Sediment	Original	RF1	RF2	RF3
Chemcial used	None	Acetic acid	Acetic acid, NH_2_OH.HCl	Acetic acid, NH_2_OH.HCl, H_2_O_2_
Metal fraction removed	None	Acid extractable fraction	Acid extractable and resducible fractions	Acid extractable, reducible, and oxidizable fractions
Metal fraction left in the sediment	Acid extractable, reducible, oxidizable, and residual fractions	Reducial, oxidizable, and residual fractions	Oxidizable and residual fractions	Residual fraction

### Sediment washing treatment

To probe the influences of different washing agents on metal fractions left in the washed sediments, every 10.0 g of the original sediment, RF1, RF2 and RF3 samples, was mixed with 100 ml of 1 M HCl, or 0.01 M EDTA, or 0.5 M H_2_O_2_ solution in a 250-ml glass bottle by a rotary shaker of 90±5 rpm for 2 h. Then the washing solution and the sediments were separated by centrifuging at 5,000 rpm for 10 min. Heavy metals of concern (i.e. Cu and Zn) in the supernatant were measured by Optima 3000XL inductively coupled plasma-atomic emission spectrometer (Perkin Elmer, USA) using USEPA Method 6010C. The separated sediment sample was flushed with deionized water to completely remove the dissociated metals.

### Heavy metal fraction and bioavailability analysis

In order to investigate the heavy metal fraction distribution affected by washing treatment with different agents, metal fractions in the washed sediment samples were determined by a modified three step BCR sequential extraction scheme [25].

As the treated sediment may be beneficially used by blending with farming soil and applying for plant growth, the metal bioavailability in the treated sediment was also assessed. The original sediment and those washed ones are slightly acidic, so the DTPA extraction test may not suitable, because it was originally developed for the neutral and near-calcareous soil with insufficient transition metals [25]. Although the EDTA extraction method may be suitable for these acidic sediment [26], EDTA is a strong chelating reagent, and often results in relatively lower correlation coefficients between the EDTA extractable metals and metals in plant roots. Therefore, the CaCl_2_ extraction test, by exchanging Ca with metals in the exchangeable complexes, was used in this study to assess the bioavailability of sediment metals. It can provide the needed data of immediately bioavailable metal content as well as the buffering capacity of the sediment, and is already widely applied in Europe, USA, New Zealand, and Australia [27]. In addition, the Diffusive Gradients in Thin-films (DGT) is a promising tool in the assessment of bioavailable fraction of metals in soil and sediment, through mimicking the uptake of metals by the plant roots [28]. Therefore, the DGT was used for the original and washed sediments, following the procedures given by Zhang et al. [28].

### Quality control and statistical analysis

The Cu, and Zn concentrations in sediments were determined by digesting 0.5 g of the soil samples (oven-dry weight) with the HCl–HNO_3_–HClO_4_–HF mixture followed by heavy metal determination. The concentrations of these metals in all the solutions were measured by Optima 3000XL inductively coupled plasma-atomic emission spectrometer (Perkin Elmer, USA) using USEPA Method 6010C, with 0.1 mg L^–1^ of the detection limits. The Cu and Zn concentrations in the CaCl_2_ extraction and TDG test were analyzed by a Z-5000 Polarized Zeeman graphite furnace Atomic Absorption Spectrometry (Hitachi, Japan) with 0.1 μg L^–1^ of the detection limits. All the reagents used in this study were of analytical grade or higher, and all the containers were soaked in 10% HNO_3_, rinsed thoroughly with deionized water, and dried before use. The geochemical standard reference sample soil in China was used to examine the precision and accuracy of acid digestion and metal determination. In this study, the recovery efficiency between the sum of the metal concentrations in individual fractions and the measured total metal concentration in the soil samples, which ranged in 97~109% for Cu and 105~113% for Zn, were calculated to check the reliability of the sequential extraction procedure.

All these experiments were performed in at least triplicate. The average, standard deviation and percentage difference were reported. The statically significant difference among different treatments was analyzed using one factor ANOVA analysis by Microsoft Excel 2013 at the 5% significance levels.

## Results and discussion

### Sediment characterization

**Fig 2:**
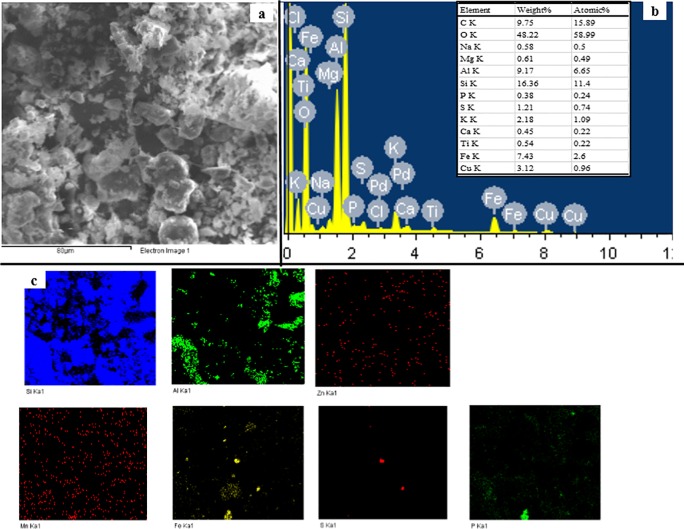
SEM-EDS-Mapping spectra of the original sediment (a: SEM spectra; b: EDS spectra; and c: element mapping spectra).

**Fig 3:**
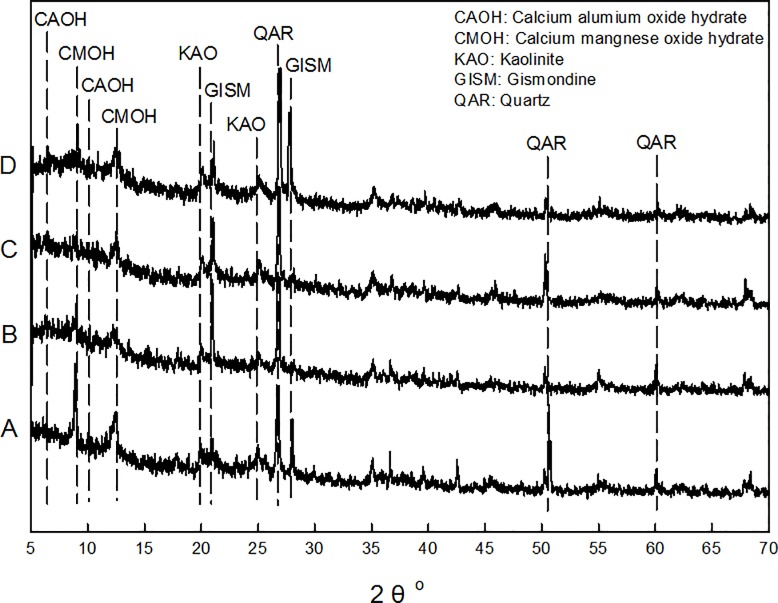
XRD spectra of a) the original sediment; and the ones washed b) HCl-washed sediment, c) EDTA-washed sediment, and d) H_2_O_2_-washed sediment.

SEM-EDS Spectra (Fig 2) showed the sediment contains high portions of Al and Si besides C and O, as alumino silicate mineral is a basic component of sediment. The XRD spectra (Fig 3) also showed the majority minerals in the original sediment included quartz, kaolinite, gismondine, and so on. No mineral containing Cu and Zn was found in the XRD spectra, likely due to their presence in amorphous forms. SEM-EDS mappings showed Fe, S and P were heterogeneously distributed. The distributions of Cu and Zn were homogeneous, and most Cu and Zn in the sediment were not associated with the components containing S and P. Cu distribution in SEM-EDS mapping seemed in a good agreement with that of Mn. BCR sequential extraction results confirmed majority of Cu (41.4%) (S1 Table) in the sediment existed as reducible fraction, which were expected to be Mn(V) or Fe(III) hydro-oxides. Therefore, most Cu may be associated with the components containing Mn(V) in the sediment.

Despite the sediment was found to have a higher level Zn than Cu (630.5±29.5 mg kg^-1^ vs. 190.5±11.2 mg kg^-1^), SEM-EDS spectra could not quantitatively determine Zn. It may be ascribed to the wider distribution of Zn in the sediment, which diluted Zn concentration in the visible region covered by SEM-EDS. In fact, BCR sequential extraction results indicated that most Zn (54.1%) (S1 Table) was found in acid extractable fraction, which preferred to be associated with the components such as oxides, carbonates and so on.

### Metal removal by chemical washing

**Fig 4:**
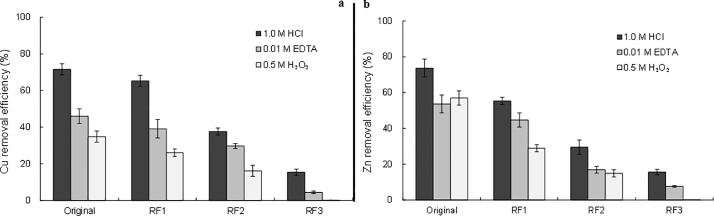
a) Cu, and b) Zn removal efficiencies in the original sediment and different extraction samples after washing by 1.0 M HCl, 0.01 M EDTA, and 0.5 M H_2_O_2._

Fig 4 illustrates Cu and Zn removal efficiencies in original sediment and different extraction samples after washing by HCl, EDTA and H_2_O_2_. A little bit higher Zn removal efficiency than Cu was observed in the original sediment. It’s because more Zn is present in the acid extractable fraction than Cu in the original sediment. Metal removal capacity of these three agents was found generally in the order: HCl > EDTA > H_2_O_2_ in most sediment samples. Except that H_2_O_2_ resulted in a higher Zn removal in the original sediment sample than EDTA, it is likely due to the relatively weak binding between EDTA and Zn (pK_Zn-EDTA_ = 16.5; pK_Cu-EDTA =_ 18.8, [18]). These results are different from what reported by Yoo et al. [23] as well as Udovic and Lestan [29], both of whom applied EDTA and HCl in soil or sediment and found EDTA was more effective in terms of extracting capacity. The difference may be explained the higher carbonate content in marine sediment [23] and in calcareous soil [30] can buffer the H^+^ attack during HCl washing, leading to a lower metal removal.

As shown in Fig 4, Cu and Zn removal efficiencies in the original sediment was found higher than those in RF1 and RF2 with the same washing agent, and the lowest removal efficiency was recorded in RF3. As most labile metal species were removed in each step of extraction following the BCR procedures, stability of Cu and Zn bound in sediment became stronger successively in the order of the original sediment, RF1, RF2, and RF3, though some metal species might be reabsorbed and re-distributed [30]. Metal removal efficiencies correlated with the binding strength between metals and sediment, and a stronger binding resulted in a diminished removal.

### Metal species transformation during washing

**Fig 5:**
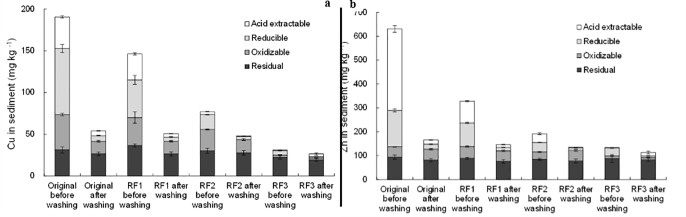
Fraction distribution of a) Cu, and b) Zn in the original sediment and different extraction samples after washing by 1.0 M HCl.

**Fig 6:**
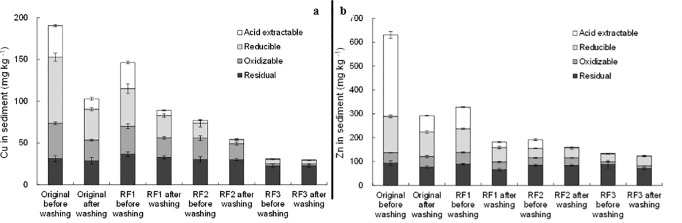
Fraction distribution of a) Cu, and b) Zn in the original sediment and different extraction samples after washing by 0.01 M EDTA.

**Fig 7:**
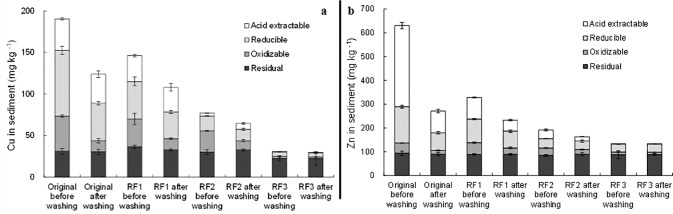
Fraction distribution of a) Cu, and b) Zn in the original sediment and different extraction samples after washing by 0.5 M H_2_O_2_.

Cu and Zn contents in the sediment before and after washing with HCl, EDTA and H_2_O_2_ are listed in Table 2. The corresponding metal fraction distributions are presented in Figs 5–7. Metal fraction distributions in the pretreated samples (RF1, RF2, and RF3) seemed not consistent with what is initially defined. There were still considerable acid extractable species in RF1 samples (Cu 21.3%, and Zn 27.8%), some reducible and acid extractable ones in RF2 samples (Cu 23.1% + 4.6%, and Zn 20.5% + 19.0%), and oxidizable and reducible species in RF3 samples (Cu 8.1% + 17.1%, and Zn 8.0% + 24.8%) (S1 Table). It shall be because some metal species were reabsorbed or redistributed during the BCR extraction from the strongly-bound fractions to weakly-associated ones, as reported in the previous studies [31]. It also confirmed that metal fraction distribution obtained by the BCR sequential extraction procedures was only operationally defined, indirectly suggesting changes of the metal species and binding strength.

**Table 2:** Cu and Zn contents in the original sediment and different extraction samples before and after chemical washing. **(**The value in parentheses are the percentage difference based on the mean value.).

Metal	Sample	Before washing	After HCl washing	After EDTA washing	After H_2_O_2_ washing
Cu (mg kg^-1^)	Original sediment	190.5±15.2 (15.9%)	54.1±0.3 (0.4%)	102.9±2.8 (2.5%)	124.1±15.1 (24.2%)
	RF1	146.1±9.3 (12.7%)	50.7±2.0 (7.7%)	89.0±4.6 (10.1%)	108.0±1.9 (3.1%)
	RF2	77.1±12.2 (29.4%)	48.0±1.2 (4.8%)	54.2±3.8 (6.5%)	64.7±4.7 (14.5%)
	RF3	31.1±0.2 (1.2%)	26.3±1.0 (7.6%)	29.7±1.5 (9.8%)	29.7±1.1 (6.7%)
Zn (mg kg^-1^)	Original sediment	630.5±12.7 (3.8%)	165.8±4.2 (4.6%)	291.9±7.0 (2.3%)	271.6±35.4 (22.9%)
	RF1	328.1±17.2 (9.1%)	146.3±3.4 (4.6%)	181.4±5.3 (3.2%)	233.3±6.9 (5.6%)
	RF2	191.9±22.2 (23.1%)	135.3±6.3 (8.1%)	159.5±12.0 (15.0%)	188.1±3.8 (4.0%)
	RF3	134.2±1.2 (1.7%)	113.1±9.3 (15.8%)	124.0±10.5 (15.6%)	134.0±1.5 (2.0%)

### HCl washing

As illustrated in Fig 5, HCl washing was found to remove majority of acid extractable and reducible Cu and Zn. There was no statistically significant difference in the amounts of remaining Cu and Zn in the original sediment, RF1, and RF2 samples after HCl washing (*p* = 0.57 for Cu, and *p* = 0.27 for Zn in one factor ANOVA analysis by Microsoft Excel 2013). So, Cu and Zn removal by 1.0 M HCl washing showed no appreciable influence from the extraction pretreatment, which was used to sequentially remove acid extractable and reducible Cu and Zn prior to chemical washing. XRD spectra (Fig 3B) showed the disappearance or diminish of some mineral peaks, such as calcium manganese oxide hydrate and calcium aluminum oxide hydrate after HCl washing. H^+^ attack and destroy of hydro(oxides) in the sediment, which primarily hosts acid extractable and reducible metal species, are the predominant mechanisms for HCl washing [32]. It well demonstrated the effective capacity of HCl solution for removing metals from sediment.

HCl washing was also found to partially remove oxidizable Cu, but seemed not play a pronounced role in the removal of oxidizable Zn (Fig 5). It may be due to the lower percentage of oxidizable Zn than Cu in the original sediment (S1 Table). Acidification is the dominant driving factor for the release of metal sulfides [33], which is widely considered as the oxidizable species. In fact, acid volatile sulfides (AVS), widely used to predict the acute metal toxicity in sediment, is operationally defined based on what can be mobilized from the sediment by treatment with 0.5 M HCl for 1 h [34].

HCl washing slightly decreased residual Cu and Zn in original sediment, RF1, RF2 and RF3 samples (Fig 5, and S1 Table). In RF3 samples, an obvious increase in acid extractable Cu and Zn was found, indicating the transformation of residual to acid extractable fraction by HCl washing as reported previously [35, 36]. These findings consistently confirmed that residual metal species can be released through destroying crystal mineral structures [35]. However, the breakdown of the crystal mineral structure is a kinetic process. Incomplete breakdown may in turn result in increase of metal mobility and bioavailability.

### EDTA washing

As illustrated in Fig 6, unlike HCl, the amounts of remaining Cu and Zn in the studied samples followed an order of original sediment > RF1 > RF2 > RF3 after EDTA washing, though EDTA washing was found to significantly remove acid extractable and reducible Cu and Zn. This may be ascribed to the extraction pretreatment of the sediment, which had facilitated in removing Cu and Zn from the sediment prior to EDTA washing. Compared with HCl, EDTA had less capacity to remove acid extractable and reducible (especially reducible) Cu and Zn. Similar to HCl, EDTA showed a higher capacity to remove the oxidizable Cu than Zn. EDTA washing also slightly decreased the residual species of Cu and Zn in the washed sediment samples. It well correlated with previous reports [36, 37]. But, Cu and Zn removal from RF3 sample was more negligible after EDTA washing than HCl.

EDTA was reported to release metal species in soil or sediment through direct complexation and indirect dissolution. Direct complexation is via the formation of stable and soluble complex with labile metal species that are often present in acid extractable fraction. Whereas, indirect dissolution is to dissolve soil or sediment components, releasing the associated metals that are usually in reducible, oxidizable, and residual fractions. XRD spectra (Fig 3C) confirmed the occurrence of indirect dissolution. Some peaks of minerals such as calcium manganese oxide hydrate, and calcium aluminum oxide hydrate disappeared or became diminished. Acid extractable Cu in the original sediment was 37.9 mg kg^-1^ (S2 Table), which could theoretically be removed via direct complexation in the 2h long EDTA washing. The remaining acid extractable Cu in the washed sediment was considered to be resulted from the transformation of other binding fractions. In comparison, the total decrement of Cu in reducible, oxidizable and residual fractions in the original sediment was 62.2 mg kg^-1^ (S2 Table), which shall be resulted from indirect dissolution. Thus, in the original sediment, the contribution of indirect dissolution to Cu removal surpassed that of direct complexation. This finding was well consistent with Ferraro et al. [38].

In terms of Zn, its removal by EDTA in the original sediment through direct complexation shall be greater than 272 mg kg^-1^ (the difference of acid extractable Zn before and after EDTA washing) (S2 Table). Zn removal by EDTA indirect dissolution was less than 65.8 mg kg^-1^, corresponding to the decrement of Zn in reducible, oxidizable, and residual fractions. This indicated that direct complexation of Zn-EDTA dominated Zn removal, although EDTA had a stronger affinity with Cu than Zn. The relatively weaker binding of Zn with the sediment (54.1% of Zn vs. 19.9% of Cu in acid extractable fraction) may well explain these distinct results of Cu and Zn removal by EDTA washing.

An increase of reducible Zn (40.7±2.8 mg kg^-1^ vs. 33.3±1.5 mg kg^-1^) and a decrease of residual Zn (70.4±4.1 mg kg^-1^ vs. 88.3±17.1 mg kg^-1^) were observed in the washed RF3 sample (Fig 6B, and S2 Table), although only 7.6% of the total Zn was removed. It suggested that the transformation of non-labile Zn to easily extractable fraction should have occurred, possibly altering the bioavailability and toxicity of the remaining metal species in the washed sediment. This is consistent with many previous studies [22, 36, 39, 40, 41, 42].

### H_2_O_2_ washing

As shown in Fig 7, Cu and Zn in oxidizable fraction were found remarkably decreased during H_2_O_2_ washing in all sediment samples. Especially for Cu, it had a higher portion of oxidizable fraction than Zn in the original sediment (Cu 22.1% vs. Zn 6.8%). The corresponding removal efficiency of oxidizable Cu was much higher than those of other fractions, given the transformation among metal fractions was excluded. In fact, the transformation from residual fraction to oxidizable one by H_2_O_2_ can be negligible, as the amount of residual Cu and Zn seemed not significantly altered (S3 Table). The deceases of Cu and Zn in oxidizable fraction in the original sediment after H_2_O_2_ washing were greater or close to those in EDTA and HCl washing, respectively. Whereas, H_2_O_2_ washing obtained lower total Cu and Zn removal than EDTA and HCl. It indicated that H_2_O_2_ had a much stronger selectivity over certain metal binding fractions than EDTA and HCl, which preferentially released Cu and Zn species according to their binding strength.

AVS content in the original sediment was 15.6 μmol g^-1^, and the total molarity of oxidizable Cu and Zn was 1.33 μmol g^-1^. Therefore, considerable oxidizable Cu and Zn can exist as sulfides. After H_2_O_2_ washing, AVS decreased to 0.2 μmol g^-1^. So, the sulfide oxidization might well explain the release of Cu and Zn [34, 43]. Meanwhile, acid extractable Cu in RF2 sample was observed obviously increased after H_2_O_2_ washing (S3 Table), suggesting the potential transformation from oxidizable Cu to acid extractable one, as reported by Yoo et al. [44, 45]. In addition, the light acidity of added H_2_O_2_ itself (pH 4.7) can also lead to the removal of some acid extractable metal species.

### Metal bioavailability in the treated sediment

The bioavailable levels of Cu and Zn in the original sediment and the washed samples were assessed by CaCl_2_ extraction and DTG test. The results are listed in Table 3. HCl washing apparently increased bioavailable levels of Cu and Zn (*p = 0*.*013 or 0*.*0004* for Cu and 0.020 or 0.0006 for Zn in CaCl2 extraction or DTG test in one-way ANOVA analysis). As discussed above, HCl can significantly destroy and dissolve sediment components, and the process is rate limiting. Instead of metal removal, the incomplete breakdown of sediment components could enhance the transformation of reducible and oxidizable fractions to acid extractable fraction which is typically bioavailable.

**Table 3:** Bioavailability of Cu and Zn in the sediment assessed by CaCl2 extraction and diffusive gradients in thin-films. (The values in parentheses are the percentage differences based on the mean.).

	CaCl_2_ extraction		DTG test	
	Cu (μg L^-1^)	Zn (μg L^-1^)	Cu (μg L^-1^)	Zn (μg L^-1^)
Oringial sediment	145±3 (4.1%)	155±14 (16.3%)	16.1±1.2 (13.0%)	23.8±0.9 (7.6%)
Sediment washed with 1 M HCl	261±47 (34.5%)	281±56 (39.9%)	51.6±5.6 (21.1%)	65.6±7.4 (22.2%)
Sediment washed with 0.01 M EDTA	163±30 (34.9%)	145±35 (42.1%)	11.6±1.3 (21.6%)	25.6±2.9 (21.1%)
Sediment washed with 0.5 M H_2_O_2_	190±15 (15.5%)	143±15 (10.7%)	11.1±0.9 (16.2%)	23.6±1.4 (11.0%)

In comparison, EDTA and H_2_O_2_ washing seemed not significantly influence (EDTA washing: *p = 0*.*36* or for Cu, and *p = 0*.*66 or* for Zn in CaCl_2_ extraction analyzed by one factor ANOVA analysis; H_2_O_2_ washing: p = 0.36 for Zn in CaCl_2_ extraction analyzed by one factor ANOVA analysis) the bioavailability of Zn and Cu remaining in the washed sediment. EDTA directly chelated with acid extractable metal species, part of which is often considered bioavailable. Consequentially, it can offset the increment in metal bioavailability induced by the destabilized metal species that was resulted from the incomplete detachment during the EDTA-enhanced dissolution process. In addition, H_2_O_2_ washing can remove Cu and Zn in the sediment primarily by oxidizing sulfides, with which metal species associated were widely considered not bioavailable [33].

## Conclusion

This study investigated Cu and Zn removal performance in river sediment by ex situ washing with three types of agents, i.e. HCl, EDTA, and H_2_O_2_. The associated potential metal fraction transformation was examined. The bioavailability of these metals left in the washed sediment was also assessed. HCl obtained the highest Cu and Zn removal through destroying the reducible, oxidizable and residual sediment components. Meanwhile it apparently transformed metal fractions to acid extractable one, resulting in an increase of metal bioavailability. Therefore, the feasibility of washing with HCl for sediment remediation should be reconsidered due to the caused high metal bioavailability. EDTA was capable of removing metals via direct complexation of labile metal species and indirect dissolution of reducible and oxidizable sediment components, where the transformation of corresponding metal binding fraction may occur. The bioavailable Cu and Zn levels in the sediment washed by EDTA seemed not increase. H_2_O_2_ obtained the lowest total Cu and Zn removal, but it preferentially removed the oxidizable metal species by oxidizing sulfides in the sediment. To maintain a good balance between labile metal species removal and avoiding increase of metal bioavailability, EDTA and H_2_O_2_ are promising additives for metal removal by sediment washing. Besides the types of washing agents employed, the influences of washing duration as well as the aging effect after washing also necessitate further investigations.

## Supporting information

S1 Table Changes of metal content in different fraction in original sediment, RF1, RF2, and RF3 samples before and after washing by 1.0 M HCl. (Uncertainties showed in the table are standard deviations with sample size n = 3 and all concentrations are expressed on a dry weight basis.)
(DOCX)

S2 Table Changes of metal content in different fraction in original sediment, RF1, RF2, and RF3 samples before and after washing by 0.01 M EDTA. (Uncertainties showed in the table are standard deviations with sample size *n* = 3 and all concentrations are expressed on a dry weight basis.)
(DOCX)

S3 Table Changes of metal content in different fraction in original sediment, RF1, RF2, and RF3 samples before and after washing by 0.5 M H_2_O_2_. (Uncertainties showed in the table are standard deviations with sample size n = 3 and all concentrations are expressed on a dry weight basis.)
(DOCX)
