# Supplementary material for: Metal removal and associated binding fraction transformation in contaminated river sediment washed by different types of agents
Source: PLoS One. 2017 Mar 28;12(3):e0174571. doi: 10.1371/journal.pone.0174571 (PMC5370133; doi:10.1371/journal.pone.0174571)
Supplement: S1 Table — (Uncertainties showed in the table are standard deviations with sample size n = 3 and all concentrations are expressed on a dry weight basis.) (DOCX) [file pone.0174571.s001.docx]

**S1 Table Changes of metal content in different fraction in original sediment, RF1, RF2, and RF3 samples before and after washing by 1.0 M HCl** (Uncertainties showed in the table are standard deviations with sample size *n*=3 and all concentrations are expressed on a dry weight basis)

| Metal removal by 1.0 M HCl washing | | Cu (mg kg^-1^) | |  | Zn (mg kg^-1^) | |
| --- | --- | --- | --- | --- | --- | --- |
|  |  | Before washing | After washing |  | Before washing | After washing |
| Original sediment | Acid extractable | 37.9±1.2 | 5.7±0.4 |  | 341.1±13.7 | 17.7±1.2 |
|  | Reducible | 78.9±4.7 | 6.7±0.5 |  | 152.2±5.8 | 20.5±1.6 |
|  | Oxidizable | 42.2±1.4 | 15.1±1.1 |  | 43.1±1.1 | 46.2±3.2 |
|  | Residual | 31.2±3.6 | 26.5±2.1 |  | 93.8±8.9 | 81.2±6.4 |
|  |  |  |  |  |  |  |
| RF1 sample | Acid extractable | 31.1±1.5 | 4.1±0.1 |  | 91.1±2.3 | 12.1±1.4 |
|  | Reducible | 45.1±5.3 | 4.9±0.4 |  | 98.5±2.7 | 14.2±1.4 |
|  | Oxidizable | 33.3±6.9 | 15.2±0.9 |  | 49.3±2.7 | 43.9±3.1 |
|  | Residual | 36.5±1.8 | 26.3±2.5 |  | 89.2±3.1 | 76.1±7.2 |
|  |  |  |  |  |  |  |
| RF2 sample | Acid extractable | 3.5±0.3 | 0.7±0.1 |  | 36.5±4.1 | 2.0±0.3 |
|  | Reducible | 17.8±0.4 | 3.8±0.1 |  | 39.4±1.1 | 10.7±1.7 |
|  | Oxidizable | 25.4±0.7 | 15.7±1.1 |  | 30.7±2.1 | 44.4±3.2 |
|  | Residual | 30.2±2.8 | 27.7±2.8 |  | 85.1±4.1 | 78.0±8.1 |
|  |  |  |  |  |  |  |
| RF3 sample | Acid extractable | 0.8±0.1 | 3.2±1.4 |  | 1.6±0.1 | 13.9±6.2 |
|  | Reducible | 5.3±0.3 | 1.7±0.2 |  | 33.3±1.5 | 7.3±1.1 |
|  | Oxidizable | 2.5±0.8 | 1.9±0.4 |  | 10.7±1.7 | 8.5±2.1 |
|  | Residual | 22.4±2.2 | 19.3±1.5 |  | 88.3±17.1 | 83.2±6.6 |
