# Supplementary material for: Metal removal and associated binding fraction transformation in contaminated river sediment washed by different types of agents
Source: PLoS One. 2017 Mar 28;12(3):e0174571. doi: 10.1371/journal.pone.0174571 (PMC5370133; doi:10.1371/journal.pone.0174571)
Supplement: S3 Table — (Uncertainties showed in the table are standard deviations with sample size n = 3 and all concentrations are expressed on a dry weight basis.) (DOCX) [file pone.0174571.s003.docx]

**S3 Table Changes of metal content in different fraction in original sediment, RF1, RF2, and RF3 samples before and after washing by 0.5 M H_2_O_2_** (Uncertainties showed in the table are standard deviations with sample size *n*=3 and all concentrations are expressed on a dry weight basis)

| Metal removal by 1.0 M HCl washing | | Cu (mg kg^-1^) | |  | Zn (mg kg^-1^) | |
| --- | --- | --- | --- | --- | --- | --- |
|  |  | Before washing | After washing |  | Before washing | After washing |
| Original sediment | Acid extractable | 37.9±1.2 | 35.1±4.1 |  | 341.1±13.7 | 90.9±7.3 |
|  | Reducible | 78.9±4.7 | 45±2.1 |  | 152.2±5.8 | 74.1±5.1 |
|  | Oxidizable | 42.2±1.4 | 13.4±2.7 |  | 43.1±1 | 16.1±2.0 |
|  | Residual | 31.2±3.6 | 30.4±3.1 |  | 93.8±8.9 | 89.9±6.7 |
|  |  |  |  |  |  |  |
| RF1 sample | Acid extractable | 31.1±1.5 | 29.5±4.4 |  | 91.1±2.3 | 46.7±3.1 |
|  | Reducible | 45.1±5.3 | 32.0±1.8 |  | 98.5±2.7 | 70.1±5.3 |
|  | Oxidizable | 33.3±6.9 | 13.4±1.1 |  | 49.3±2.7 | 26.7±3.4 |
|  | Residual | 36.5±1.8 | 32.9±1.1 |  | 89.2±3.1 | 89.6±3.5 |
|  |  |  |  |  |  |  |
| RF2 sample | Acid extractable | 3.5±0.3 | 6.9±1.0 |  | 36.5±4.1 | 18.1±0.9 |
|  | Reducible | 17.8±0.4 | 13.9±1.3 |  | 39.4±1.1 | 35.4±4.7 |
|  | Oxidizable | 25.4±0.7 | 10.9±1.3 |  | 30.7±2.1 | 19.9±2.1 |
|  | Residual | 30.2±2.8 | 32.8±1.1 |  | 85.1±4.1 | 89.6±6.7 |
|  |  |  |  |  |  |  |
| RF3 sample | Acid extractable | 0.8±0.1 | 0.4±0.2 |  | 1.6±0.1 | 18.1±0.9 |
|  | Reducible | 5.3±0.3 | 4.8±0.9 |  | 33.3±1.5 | 35.4±4.7 |
|  | Oxidizable | 2.5±0.8 | 1.9±0.5 |  | 10.7±1.7 | 19.9±2.1 |
|  | Residual | 22.4±2.2 | 22.4±8.1 |  | 88.3±17.1 | 89.6±6.7 |
